# Mediators of the association between childhood body mass index and educational attainment: Analysis of a UK prospective cohort study

**DOI:** 10.1111/ijpo.13014

**Published:** 2023-02-23

**Authors:** Kirsty Bowman, Tim Cadman, Ana Goncalves Soares, Oliver Robinson, Amanda Hughes, Jon Heron, Alexa Blair Segal, Maria Carmen Huerta, Laura D. Howe

**Affiliations:** ^1^ MRC Integrative Epidemiology Unit University of Bristol Bristol UK; ^2^ Population Health Sciences University of Bristol Bristol UK; ^3^ Department of Public Health University of Copenhagen Copenhagen Denmark; ^4^ MRC Centre for Environment and Health School of Public Health, Imperial College London London UK; ^5^ Centre for Health Economics & Policy Innovation Imperial College Business School London UK

**Keywords:** ALSPAC, body mass index, children, education attainment, GCSEs, mediation

## Abstract

**Background:**

Higher body mass index (BMI) in childhood is associated with lower academic achievement.

**Objective:**

To explore potential pathways linking childhood BMI with educational attainment.

**Methods:**

Using data from the Avon Longitudinal Study of Parents and Children prospective cohort study (*N* = 6949), we assessed the association between BMI *z*‐scores at 11.7 years and educational attainment at 16 (General Certificate of Secondary Education [GCSE] results). Depressive symptoms, externalizing behaviours, bullying and school enjoyment were considered as potential mediators. Mediators were examined individually and jointly using sequential causal mediation.

**Results:**

Higher BMI *z*‐scores were associated with lower GCSE scores (females *β* = −3.47 95% CI −5.54, −1.41 males *β* = −4.33 95% CI −6.73, −1.94). Together, bullying, externalizing symptoms, depressive symptoms and school enjoyment mediated 41.9% of this association in females, and 23.3% in males. In males, evidence for mediation was weak (confidence intervals for all indirect effects spanned the null). In both females and males, most of the mediation was driven by externalizing symptoms.

**Conclusions:**

The detrimental effect of higher BMI on educational attainment appears to be partly explained by externalizing behaviours, particularly in females. Interventions to support behavioural problems may help the academic achievement of children with a higher body weight.

## 1 INTRODUCTION

Globally, the proportion of children and adolescents with obesity has increased dramatically over the past four decades. Between 1975 and 2016, the age standardized prevalence of obesity increased from 0.7% to 5.6% for females and from 0.9% to 7.8% for males aged 2–19 years.^1^ Children with obesity are more likely to suffer from adverse health consequences both in the shorter and longer terms including increased cardiovascular risk factors, cardiovascular disease in later life, and premature mortality.^2^, ^3^, ^4^ Moreover, children with obesity are at an elevated risk of transitioning into adulthood with obesity.^5^

There is accumulating evidence suggesting that children with obesity are more likely to experience adverse social and economic outcomes.^6^, ^7^, ^8^ Educational attainment is a particular outcome of interest as it is crucial for employment prospects, later health, well‐being and functioning in society.^9^, ^10^ Recently, a systematic review focusing on studies using more robust causal inference approaches (i.e., studies which utilized longitudinal data or methods to minimize reverse causation and unobserved confounding, such as Mendelian randomization) concluded that childhood obesity hinders educational attainment, with larger negative associations observed for females compared to males.^8^ The review focused on high‐income countries (including the United States, Taiwan, United Kingdom, Australia, Canada) and children up to the age of 18 years at baseline.^8^

Several pathways have been suggested which may link childhood obesity to lower educational attainment. Childhood and adolescent obesity has been reported to be associated with attention problems and externalizing behaviours,^11^, ^12^ depressive symptoms (especially for females) and major depressive disorder,^13^, ^14^, ^15^ which could weaken motivation and reduce educational performance.^16^, ^17^, ^18^, ^19^ Furthermore, children with obesity may be more likely to have impaired peer relationships, to be bullied and to report being socially isolated.^20^, ^21^, ^22^ These factors may reduce both their functioning within their school environment and their academic attainment; for instance, biases from teachers may result in lower teacher‐assessed grades.^23^, ^24^, ^25^, ^26^ Children and adolescents with obesity may have impaired school experiences, lower school enjoyment, disengagement and lower participation in learning activities, and lower motivation at school.^16^, ^24^, ^27^

Elucidating the pathways linking obesity to educational attainment may help to inform strategies to improve the school experience and educational attainment of children with obesity. Overall, there has been a limited number of studies examining potential pathways between childhood obesity and educational attainment.^28^, ^29^ Evidence has shown that the following factors may have a mediating role: weight‐based teasing,^24^ behavioural engagement^27^ and bullying.^30^ Furthermore, anxiety, depression and attention deficit/hyperactivity disorder (ADHD) have been shown to be of importance for educational achievement among children and adolescents with obesity.^31^ A previous analysis using the Avon Longitudinal Study of Parents and Children (ALSPAC) reported that there was no evidence for depressive symptoms (reported at 11 years), intelligence quotient (IQ) (reported at 8 years) or age at menarche mediating the relationship between body mass index (BMI) at 11 years and educational attainment at 16 years.^32^ Here, we extend on previous research by exploring other potential mediators and use g‐computation which can examine the combined effects of multiple mediators and handle intermediate confounders (e.g., bullying may be a confounder of the association between depression and educational attainment, and in itself be caused by obesity).^33^, ^34^ We used data from ALSPAC to explore potential pathways between childhood BMI and educational attainment. In particular, we sought to understand the mediating roles of depressive symptoms (assessed at a later age compared to the previous ALSPAC analysis by Booth et al.,^32^ since depressive symptoms tend to increase through adolescence^35^), bullying, externalizing behaviours (hyperactivity and conduct problems) and school enjoyment, and we explored the sex differences in these pathways. These putative mediators were chosen due to previously reported and plausible associations with childhood BMI and educational attainment, and the availability of these measures in ALSPAC. We firstly aimed to examine each mediator individually, accounting for intermediate confounders. Secondly, as the pathways may not act independently, we aimed to examine the combined contribution of the mediators based on a proposed causal ordering between the mediators using sequential causal mediation (see Figure S2).

## 2 METHODS

### 2.1 Study participants

This analysis used data from the ALSPAC which is a birth cohort within the South West of England. Pregnant women were invited to take part in the study if they were resident in the former Avon area with an estimated delivery date between 1 April 1991 and 31 December 1992. The core sample comprises 14 541 pregnant women with 13 988 children alive at 12 months. There have been regular follow‐ups via questionnaires and clinical assessment of the children and parents since the initiation of the cohort. Further detailed descriptions of the cohort are given elsewhere.^36^, ^37^ The study website contains details of all the data which is available through a fully searchable data dictionary and variable search tool (http://www.bristol.ac.uk/alspac/researchers/our-data/). Ethics approval for the study was obtained from the ALSPAC Ethics and Law Committee and the local research ethics committees.

### 2.2 Exposure: Body mass index

Children attended a clinical assessment centre at mean age 11.7 years (SD 0.24 years). Height was measured, where possible without shoes, using a Harpenden Stadiometer and recorded to the last complete millimetre. Weight was measured, where possible in underclothes and without shoes, using a Tanita Body Fat Analyser (Model TBF 305) to the nearest 50 grammes. The BMI measures, calculated as weight in kilogrammes divided by height in metres squared, were age‐ and sex‐standardized to the 1990 UK Growth Reference (38). For descriptive purposes, we also categorized children as overweight (BMI *z*‐score ≥1.04 and <1.64, ≥85th and <94th percentile) and those with obesity (BMI *z*‐score ≥1.64, ≥95th percentile) (Table S1).^39^, ^40^

### 2.3 Potential mediators

#### 2.3.1 Depressive symptoms

Children attended a clinical assessment centre at mean age 13.8 years (SD 0.21 years). Depressive symptoms were assessed via self‐report using the short version of the Mood and Feelings Questionnaire (SMFQ), which has been validated for use with children and adolescents.^41^ Within the ALSPAC cohort, the SMFQ has been shown to have good internal validity with Chronbach's alpha ranging from 0.797 (mean age at assessment 10.65 years) to 0.915 (mean age at assessment 21.95 years).^42^ The questionnaire has 13 statements asking about symptoms experienced in the previous 2 weeks with the following choice of response options: ‘true’, ‘sometimes’ or ‘not at all’ (Tables S2 and S3). Responses for the 13 statements in this analysis were summed to provide a summary score (score possible range 0–26), with higher scores reflecting greater depressive symptoms.

#### 2.3.2 Externalizing behaviours

Externalizing behaviours were based on the mother‐rated hyperactivity and conduct subscales of the Strengths and Difficulties Questionnaire^43^ which was completed when the study children were mean age 13.2 years (SD 0.18 years). The internal validity with Cronbach's alpha has been reported to be satisfactory for the parent reported conduct subscale (0.63) and the parent reported hyperactivity subscale (0.77).^44^ Each of the two subscales consist of five statements relating to children's difficulties within the previous 6 months with the following response options: ‘not true’, ‘somewhat true’ or ‘certainly true’ (Tables S2 and S3). Responses for the 10 statements in this analysis were summed to provide a summary score (score possible range 0–20), with higher scores reflecting greater externalizing behaviours. For participants with missing data on up to three items from the scale, we pro‐rated the available data to create the score.

#### 2.3.3 School enjoyment

Children completed a questionnaire at mean age 14.2 years (SD 0.24 years). The questionnaire included 39 questions related to school experience and had several items capturing school enjoyment, for example, how much the respondent's school is a place where they enjoy what they do in class.^45^ The questions centred around the child's enjoyment of classes and school with the response options: ‘strongly agree’, ‘agree’, ‘disagree’, ‘strongly disagree’ (Tables S2 and S3). Previous work used factor analysis to identify key school enjoyment variables that were distinct from each other.^45^ A previous analysis using this questionnaire, found that there was an association between school enjoyment and educational attainment.^46^ The responses for three statements on school enjoyment were summed to provide a summary score (score possible range 0–9), with higher scores reflecting lower school enjoyment.

#### 2.3.4 Bullying

Children attended a clinical assessment centre at mean age 12.8 years (SD 0.23 years). Bullying was assessed using a modified version of the Bullying and Friendship Interview Schedule (BFIS),^47^ which has been shown to have high inter‐rater reliability.^48^ A number of previous studies have shown associations between those who are classified as peer victimized/bullied (assessed using the BFIS) to be associated with mental health, self‐harm, and well‐being.^49^, ^50^, ^51^ This analysis uses the sections on received overt (direct) bullying to establish victimization and received relational (indirect) bullying to establish relational victimization. In this analysis, we used five of the statements in the received overt bullying and four statements of the received relational bullying which have been used in previous analyses and have been consistently used at previous clinical assessment centre visits.^52^ Children were asked if they had experienced any of the events in the last 6 months and if so, follow‐up questions were asked about the frequency. Response options for the frequency were: ‘seldom (1–3 times)’, ‘frequently (>4 times)’ and ‘very frequently (>1/week)’. Children who responded that they had not experienced that event were coded as a frequency of never. In this analysis, we combined both received overt and received relational bullying and the responses were summed to provide a summary score (score possible range 0–27), with higher scores reflecting greater bullying.

### 2.4 Outcome: Educational attainment

Educational attainment was assessed using a summary measure based on General Certificate of Secondary Education (GCSE) qualifications taken at age 16, which represented the end of compulsory education for study participants. Results from GCSEs and equivalent qualifications were obtained from linkage between ALSPAC and the National Pupil Database. The summary measure, a total GCSE and equivalents ‘capped’ score, is calculated by converting qualification grades into points (e.g., an A* in one subject is worth 58 points), and calculating the total from the student's best eight subjects. This is to prevent scores being inflated by students who sit more qualifications. The possible range from 8 GCSEs is 0 to 464, but a small number of students who took more advanced qualifications early had scores between 464 and 540 (thus the overall potential range of scores is 0–540).

### 2.5 Covariates

The following variables were considered as possible baseline confounders of the association between the child's BMI and their educational attainment: maternal age at pregnancy (years), maternal smoking in pregnancy (categorized as a binary variable, yes/no), housing tenure (categorized as a binary variable, mortgaged/owned/rented privately/other vs. council rented/housing association rented), highest maternal education qualification (none or Certificate of Secondary Education; vocational; O‐level; A‐level; degree or higher), maternal occupational social class based on the UK Registrar General's occupational coding system (professional, managerial and technical; skilled non‐manual/skilled manual; partly skilled/unskilled)^53^ and parity (0 vs. ≥1). Maternal smoking, parity, social class, housing tenure and highest education were derived from questionnaires completed during the antenatal period. The inclusion of these variables was guided by the literature, their plausibility, and using a directed acyclic graph (Figure S2) and thus were considered to be a common set of confounders for the exposure‐mediator, exposure‐outcome and mediator‐outcome pathways.

### 2.6 Statistical analyses

We restricted our analysis to participants who had height and weight measured at a minimum of one research clinic visit between age 7 and age 15 (eight potential measurement occasions, with measures other than the main exposure at age 11.7 years used as auxiliary variables in multiple imputation analyses) and those who had a GCSE and equivalents capped score in the National Pupil Database (for further detail see Missing data section and for further details on the imputation model see Tables S2 and S3). We excluded participants with a missing a record of ethnic background (*n* = 1894) because we did not have sufficient auxiliary data to impute ethnicity. Our analysis therefore included 6949 participants (Figure S1).

Analyses were conducted using STATA version 16.1. Mediation analyses using g‐computation, which is based on a counterfactual framework, was used to estimate natural direct (not via the mediator(s) of interest), natural indirect (via the mediator(s) of interest) and total causal effects between childhood BMI *z*‐scores and GCSE and equivalents capped score (using STATA ‘g‐formula’ package with the mediation option).^33^, ^34^ G‐formula is able to handle intermediate confounders (confounders of the mediator‐outcome association, which are caused by the exposure) and can incorporate multiple mediators to examine their joint contribution. Potential mediators ‐ depressive symptoms, externalizing behaviours, bullying and school experience ‐ were all modelled as continuous variables using summary scores. We firstly examined each of the mediators individually using g‐formula, with intermediate confounders included as appropriate for each mediator (see Figure S2 for assumptions about the inter‐relationships between mediators). In model 1, we used bullying as the individual mediator with no intermediate confounders; in model 2, we used externalizing behaviours as the individual mediator with bullying considered as an intermediate confounder; in model 3, we used depressive symptoms as the individual mediator with bullying and externalizing behaviours considered as intermediate confounders; and in model 4, we used school enjoyment as the individual mediator with bullying, externalizing behaviours and depressive symptoms considered as intermediate confounders. Each of the models had adjustments for the baseline confounders (see covariate section). In the second part of our analysis, we examined the contribution of the mediators jointly using sequential causal mediation (models 5 to 7). In model 5, we estimated the indirect effect through bullying (this includes the pathways that act through bullying and any of its effects but excludes the pathways that act only through externalizing behaviours, depressive symptoms and school enjoyment). In model 6, we estimated the indirect effect through bullying and externalizing behaviours (this includes the pathways that act through bullying and externalizing behaviours and their effects but excludes the pathways that act only through depressive symptoms and school enjoyment). In model 7, we estimated the indirect effect through bullying, externalizing behaviours and depressive symptoms (this includes the pathways that act through bullying, externalizing behaviours and depressive symptoms and their effects, but excludes pathways that act only through school enjoyment). In model 8, we estimated the indirect effect through all potential mediators. The proportion mediated reflects the indirect effect divided by the total effect. All analyses were run separately for males and females due to prior evidence that associations of BMI with educational attainment and some of the mediators may differ by sex.^8^, ^54^ We conducted a number of sensitivity analyses including (1) restricting our analysis to participants of white ethnicity (*n* = 6660) because of the low numbers in any ethnic group other than white prevents full adjustment for potential confounding by ethnicity, (2) examining the summary scores of overt bullying and relational bullying separately in case results differed for these different forms of bullying, and (3) excluding participants who had a record documented in Key Stage 4 of having either Special Education Need (SEN) School Action or a SEN Action Plus (*n* = 715), as associations may differ in these children.

### 2.7 Missing data

To deal with missing data on the exposure, the mediators or the covariates, we used multivariate multiple imputation. We imputed up to our analytical sample of 6949 participants (i.e., those who had a minimum of one research clinic visit for height and weight measures and GCSE and equivalents capped score). Prior to imputation we combined categories where there were low cell counts (*n* < 50). Multiple imputation was used to create data sets for the females and males separately (*m* = 100). The number of imputations was guided by examining the fraction of missing information (FMI), the largest FMI being 0.73 (with recommendations that the number of imputations be at least 100 times the FMI^55^, ^56^) and examining whether the Monte Carlo errors were less than 10% of the standard error of the estimated coefficients, which they were. We used multiple chained equations (mice) to impute missing data for all the variables. The imputation model contained all the variables to be included in the mediation models and variables known to be predictors of missingness, thereby increasing the plausibility of the missing at random (MAR) assumption.^57^ For further details on the imputation model see Tables S2 and S3. We carried out mediation analysis on each of the imputed datasets and standard errors were estimated using 1000 bootstrap samples within each dataset. Estimates were then combined using Rubin's rules.^58^

## 3 RESULTS

### 3.1 Descriptive statistics

Table 1 shows the characteristics of the imputed sample. The mean BMI z‐score (using the 1990 UK reference) at age 11.7 years was 0.33 (SD 1.22) for the females and 0.44 (SD 1.22) for the males. The mean GCSE point score was 346.8 (SD 79.45) for the females and 325.2 (SD 87.74) for the males (Table 1). The distributions of the variables in the imputed sample tended to be similar to those observed in the raw data (Tables S1–S3).

**TABLE 1:** Characteristics of participants included in analysis; females (*n* = 3544) and males (*n* = 3405) using imputed data.

	Females	Males
BMI *z*‐score at 11.7 years (mean [SD])	0.33 (1.22)	0.44 (1.22)
Maternal smoking in pregnancy (%)	19.2	19.8
Maternal social class (%)		
I—professional/II—Managerial and technical	33.4	33.0
IIINM—Skilled non‐manual/IIIM—Skilled manual	46.4	48.1
IV—Partly skilled/V—Unskilled	20.2	19.0
Maternal education (%)		
Degree	13.2	13.0
A‐level	25.3	25.2
O‐level	36.2	36.5
Vocational	9.6	9.8
None/CSE	15.7	15.4
Maternal housing tenure (council rented/housing association rented) (%)	11.0	10.5
Parity (≥1) (%)	54.4	54.7
Maternal age at birth (mean [SD])	28.6 (4.53)	29.0 (4.59)
Capped GCSE point score (mean [SD])	346.8 (79.45)	325.2 (87.74)

Abbreviations: BMI, body mass index; CSE, Certificate of Secondary Education, GCSE, General Certificate of Secondary Education.

### 3.2 Total causal effect, natural direct effect and natural indirect effect of BMI on educational attainment, with mediation through the individual mediators (accounting for intermediate confounding)

Table 2 shows the total causal effect, natural direct effect, natural indirect effect and the proportion‐mediated of BMI z‐scores on GCSE scores through each of the individual mediators. Higher BMI z‐scores at age 11.7 years were associated with lower GCSE scores (TCE: β for a one standard deviation higher BMI = −3.47 95% CI −5.54, −1.41 in females; males *β* = −4.33 95% CI −6.73, −1.94) (Table 2). There was evidence of mediation by externalizing behaviours in females (NIE β = −1.53 95% CI −2.94, −0.12, proportion mediated 44.3%). The direction of the indirect effects was the same in males, however, the confidence interval was wide and included the null value (NIE *β* = −0.70, 95% CI −2.35, 0.95). There was no evidence of mediation by depressive symptoms, bullying or school enjoyment in either females or males.

**TABLE 2:** Total causal effect (TCE), natural direct effect (NDE), natural indirect effect (NIE) and proportion mediated of body mass index on educational attainment examining the individual mediators from the pooled results for the females (*n* = 3544) and males (*n* = 3405).

Mediator	Intermediate confounder(s)	TCE coeff (95% CI)	NDE coeff (95% CI)	NIE coeff (95% CI)	Proportion mediated (%)
Females					
Bullying	‐	−3.47 (−5.54, −1.41)	−3.26 (−5.33, −1.18)	−0.22 (−0.53, 0.09)	6.3
Externalizing behaviours	Bullying	−3.47 (−5.54, −1.41)	−1.94 (−4.19, 0.30)	−1.53 (−2.94, −0.12)	44.3
Depression	Bullying and externalizing behaviours	−3.47 (−5.52, −1.42)	−3.46 (−5.52, −1.41)	−0.01 (−0.26, 0.24)	0.2
School enjoyment	Bullying, externalizing behaviours and depression	−3.47 (−5.55, −1.40)	−3.51 (−5.59, −1.44)	0.04 (−0.71, 0.79)	N/A
Males					
Bullying	‐	−4.33 (−6.73, −1.94)	−4.11 (−6.52, −1.71)	−0.22 (−0.57, 0.13)	5.1
Externalizing behaviours	Bullying	−4.33 (−6.73, −1.94)	−3.63 (−6.23, −1.04)	−0.70 (−2.35, 0.95)	16.2
Depression	Bullying and externalizing behaviours	−4.33 (−6.74, −1.92)	−4.03 (−6.44, −1.63)	−0.30 (−0.74, 0.14)	6.9
School enjoyment	Bullying, externalizing behaviours and depression	−4.33 (−6.72, −1.94)	−4.29 (−6.67, −1.92)	−0.04 (−0.30, 0.22)	0.9

*Note*: Models were adjusted for maternal age at pregnancy (years), maternal smoking in pregnancy, housing tenure, highest maternal education qualification, maternal social class and parity. The proportion mediated is not calculated where there is inconsistent mediation, that is, where the indirect effect is positive and the total effect is negative. The Total causal Effect 95% confidence intervals differ slightly between the models because of the estimation procedure. Proportion mediated is the average of pooled results across imputed datasets.

### 3.3 Total causal effect, natural direct effect and natural indirect effect of BMI on educational attainment, with sequential causal mediation to account for multiple inter‐related mediators

Table 3 shows the total causal effect, natural direct effect, natural indirect effect, and the proportion‐mediated of BMI z‐scores on GCSE scores using the sequential mediation approach, which considers the joint effects of multiple mediators. For both females and males, the four mediators together accounted for a moderate proportion of the association between BMI at age 11 and educational attainment; the overall proportion mediated was 41.9% in females and 23.3% in males. Most of this effect was driven by externalizing behaviours. In males, the confidence interval for the NIE was wide and included the null value. There appeared to be some suppression when both externalizing symptoms and depressive symptoms were included in the model together, but this was reversed when school enjoyment was additionally included.

**TABLE 3:** Total causal effect (TCE), natural direct effect (NDE), natural indirect effect (NIE) and proportion mediated of body mass index on educational attainment from the sequential causation mediation from the pooled results for the females (*n* = 3544) and males (*n* = 3405).

Mediator(s)	TCE coeff (95% CI)	NDE coeff (95% CI)	NIE coeff (95% CI)	Proportion mediated (%)
Females				
Bullying	−3.47 (−5.54, −1.41)	−3.26 (−5.33, −1.18)	−0.22 (−0.53, 0.09)	6.3
+ Externalizing behaviours	−3.47 (−5.55, −1.40)	−2.40 (−4.63, −0.17)	−1.07 (−2.53, 0.39)	31.0
+ Externalizing behaviours + Depression	−3.47 (−5.52, −1.42)	−3.14 (−5.39, −0.89)	−0.33 (−1.75, 1.08)	9.6
+ Externalizing behaviours + depression + school enjoyment	−3.47 (−5.53, −1.42)	−2.03 (−4.31, 0.26)	−1.45 (−3.06, 0.16)	41.9
Males				
Bullying	−4.33 (−6.73, −1.94)	−4.11 (−6.52, −1.71)	−0.22 (−0.57, 0.13)	5.1
+ Externalizing behaviours	−4.33 (−6.72, −1.94)	−3.74 (−6.32, −1.15)	−0.60 (−2.30, 1.11)	13.8
+ Externalizing behaviours + depression	−4.33 (−6.79, −1.88)	−5.69 (−8.25, −3.12)	1.35 (−0.36, 3.07)	N/A
+ Externalizing behaviours + depression + school enjoyment	−4.33 (−6.74, −1.93)	−3.33 (−5.87, −0.78)	−1.01 (−2.67, 0.65)	23.3

*Note*: Models were adjusted for maternal age at pregnancy (years), maternal smoking in pregnancy, housing tenure, highest maternal education qualification, maternal social class and parity. The proportion mediated is not calculated where there is inconsistent mediation, that is, where the indirect effect is positive and the total effect is negative. The Total causal Effect 95% confidence intervals differ slightly between the models because of the estimation procedure. Proportion mediated is the average of pooled results across imputed datasets.

## 4 SENSITIVITY ANALYSES

The results from the sensitivity analysis restricting to those of white British ethnicity were broadly similar to those from the main analysis (Tables S4 and S5). We repeated our individual mediator analysis using the overt and relational bullying components instead of the overall bullying summary scores (Table S6). For both the males and females the direction of the indirect effect was the same as the total effect. For the females the magnitude of the indirect effect was stronger for overt bullying then relational bullying. However, we did not find any statistical evidence for mediation by either component in either females or males. The results from the sensitivity analysis excluding participants who had a record documented in Key Stage 4 of having either Special Education Need (SEN) School Action or a SEN Action Plus were broadly similar to the results from the main analysis (Tables S7 and S8).

## 5 DISCUSSION

In this cohort study, each one SD higher BMI was associated with a reduction in the GCSE score of approximately four points. This represents about 0.04 standard deviations of the score, so the magnitude of this association is small. In females, approximately 44% of the association between BMI and educational attainment was mediated by externalizing behaviours, with the equivalent proportion in males being 16%. Jointly, the four mediators we considered explained 42% of the association between BMI and educational attainment in females, and 23% in males. A considerable proportion of the association between BMI and educational attainment was not explained by the mediators we considered, potentially suggesting other pathways are important, particularly in males. Self‐esteem and school absence, key potential mediators, could not be explored due to a lack of data at the appropriate time points.

Previous analyses exploring potential pathways between BMI and educational attainment have suggested that weight‐based teasing^24^ and bullying^30^ may have a mediating role. Here, we found no evidence of a mediating role for bullying although educational attainment was assessed at an earlier age in the previous studies (9.5 years and 12 years, respectively, as opposed to 16 years in the current study). A former analysis, also using ALSPAC, examined individual mediators in relation to BMI and educational attainment, and found there was no mediation via depression for females and males.^32^ Depressive symptoms were assessed at age 11, which is earlier than the current study, and before the age at which depressive symptoms typically begin to rise most rapidly.^35^ We similarly found no evidence of depressive symptoms having a mediating role after accounting for bullying and externalizing behaviours as intermediate confounders (not part of the analysis in the previous paper).

Analyses using genetic data as instrumental variables (Mendelian randomization) have suggested that the association between BMI and educational attainment may be causal.^59^, ^60^, ^61^ However, recent studies that have applied Mendelian randomization approaches to family‐level data have suggested that biases due to family‐level processes may drive this association. These processes may include intergenerational effects, including influence of parental BMI on children's outcomes, and non‐random partnership in the parent's generation with respect to educational attainment or body weight (assortative mating).^62^ It is therefore possible that the associations observed in this study in part reflect family‐level processes, and not only the influence of a child's BMI.

### 5.1 Strengths and limitations

We used data for over 6000 children from a large birth cohort. The large sample size enabled analysis of males and females separately, given our a priori hypothesis that both the association between BMI and educational attainment and the mediating pathways may differ by sex. A strength of this analysis was using objective research clinic‐based measures of BMI and linked educational data, thereby minimizing measurement error in the exposure and outcome. Measurement error in a mediator can lead to an underestimation of the indirect effect.^63^ We attempted to control for known confounders, including intermediate confounders. However, as this was an observational study, there may still be residual confounding, including confounders of the mediator‐outcome association, which can induce collider bias.^64^ We did not include age of menarche as a baseline confounder in our analysis as the mean age at onset of menarche (mean age of onset 12.6 years)^65^ was later than the BMI measure used in this analysis. We did not include variables on neighbourhood deprivation as we used individual levels which are likely to address the confounding by social economic disadvantage. Our sequential modelling approach requires assumptions about the causal ordering of the mediators. We based our assumptions on the most likely direction of causality and the exact ages of measurement of the variables, but recognize that alternative specifications would also be plausible, and may have resulted in different results. However, we do not think the overall conclusions would change with different specifications of the causal ordering. Whilst we have temporal ordering between our exposure, mediator and outcome variables, this is a necessary, but not sufficient, criterion for causal inference.

For some of the mediators there was a high proportion of missing data, and thus we used multiple imputation to maximize statistical power and reduce selection bias. While the inclusion of confounders and auxiliary variables adds to the likelihood of the MAR assumption being plausible for multiple imputation, we cannot exclude the possibility of data being missing not at random. ALSPAC is a birth cohort study of those who were resident in the former Avon area (South West England) and thus is not nationally representative; there are higher proportions of those from higher socioeconomic status and white British ethnic groups, which could limit the generalizability of these results.^36^, ^66^

## 6 CONCLUSIONS

Higher BMI *z*‐scores at age 11.7 years were associated with lower educational attainment. The detrimental effect of higher BMI on educational attainment appears to be partly explained by externalizing behaviours, particularly in females. Interventions to support behavioural problems (conduct problems and hyperactivity) may help the academic attainment of children with a higher body weight.

## CONFLICT OF INTEREST STATEMENT

Prof. Howe reports grants from Health Foundation, grants from UK Medical Research Council, during the conduct of the study.

## Supporting information


**Data S1.** Supporting information.
