# Supplementary material for: Mediators of the association between childhood body mass index and educational attainment: Analysis of a UK prospective cohort study
Source: Pediatr Obes. 2023 Feb 23;18(5):e13014. doi: 10.1111/ijpo.13014 (PMC10909521; doi:10.1111/ijpo.13014)
Supplement: Supplementary file 1 — Data S1. Supporting information. [file IJPO-18-e13014-s001.pdf]

# Mediators of the association between childhood BMI and educational attainment: analysis of a UK prospective cohort study

Kirsty Bowman, Tim Cadman, Ana Goncalves Soares, Oliver Robinson, Amanda Hughes, Jon Heron, Alexa Blair Segal, Maria Carmen Huerta, Laura D Howe*

Affiliations

MRC Integrative Epidemiology Unit at the University of Bristol, Bristol, UK (KB, AGS, AH, JH, LDH)

Population Health Sciences, University of Bristol, Bristol, UK (KB, AGS, AH, JH, LDH)

Department of Public Health, University of Copenhagen, Copenhagen, Denmark (TC)

Centre for Health Economics & Policy Innovation, Imperial College Business School, London, UK (MCH, ABS)

MRC Centre for Environment and Health, School of Public Health, Imperial College London, UK (OR)

Correspondence

*Corresponding author: email Laura.Howe@bristol.ac.uk; postal address MRC Integrative Epidemiology Unit at the University of Bristol, Oakfield House, Oakfield Grove, Bristol BS8 2BN, UK

Figure S1: Flow chart of inclusion and exclusion of ALSPAC participants into the study sample

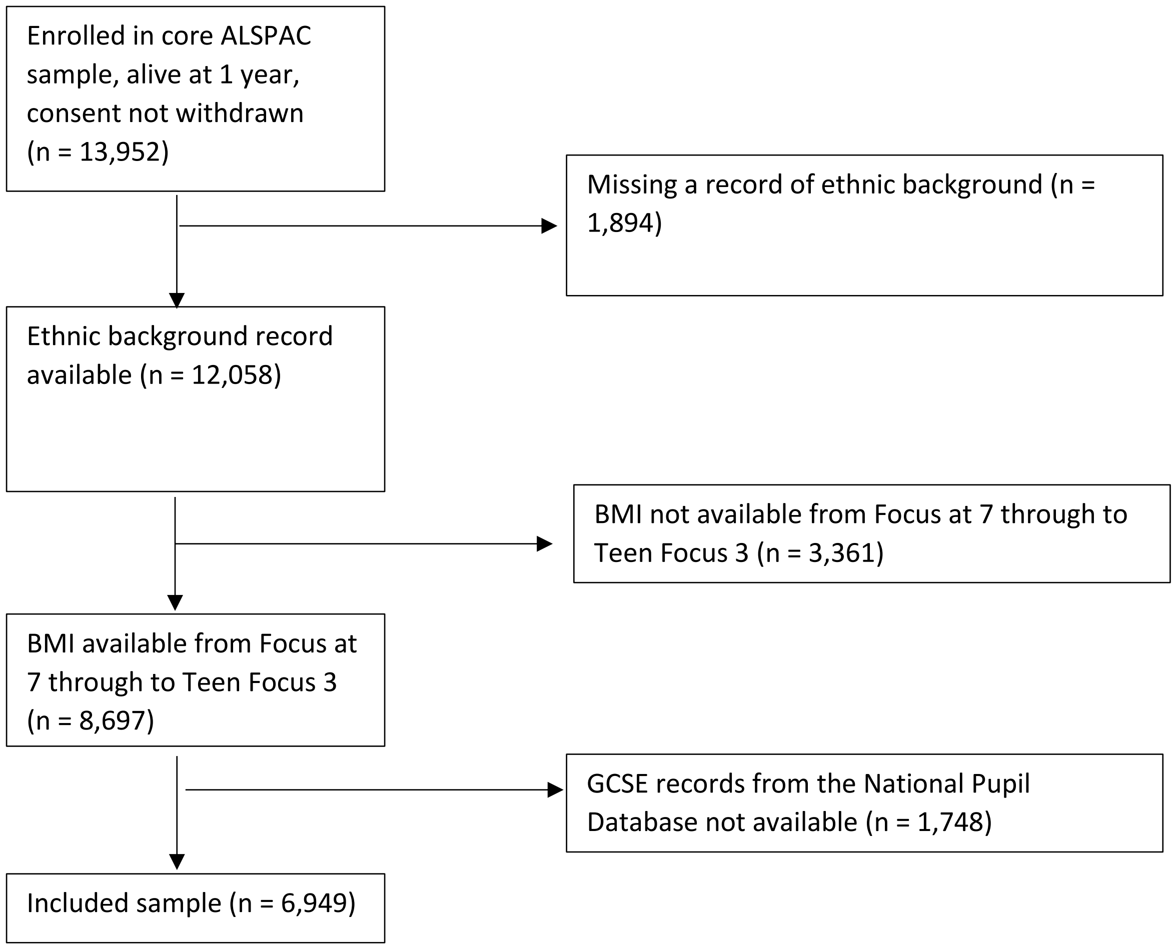

Figure S2: Directed Acyclic Graph (DAG) illustrating hypothesised relationships among exposure, outcome, and mediators

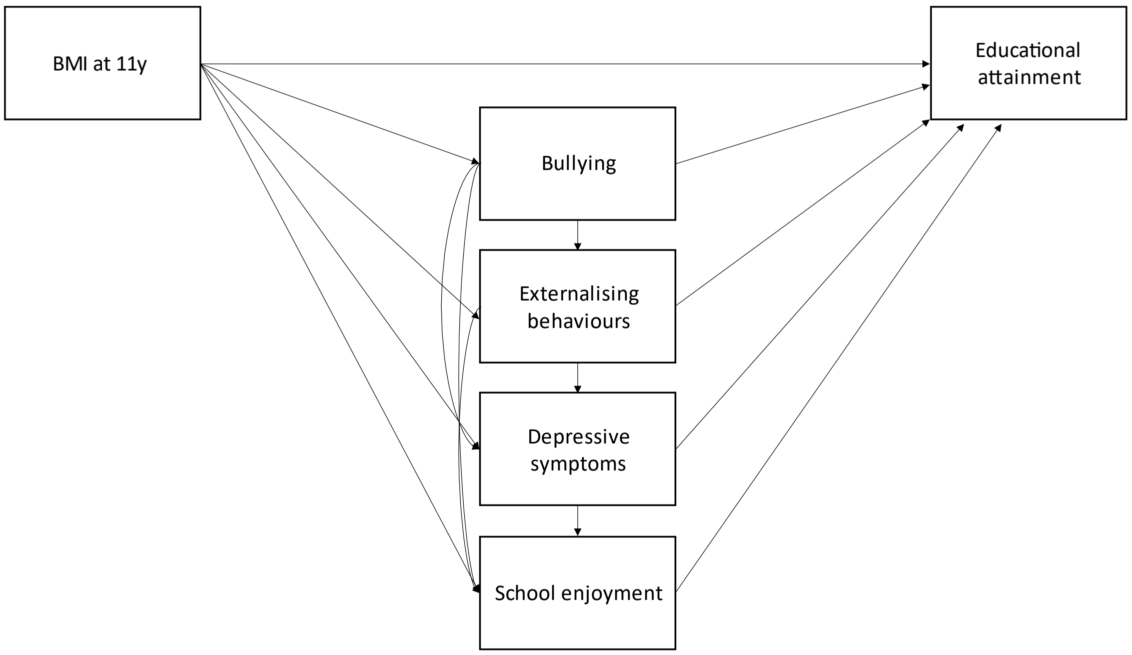


*Note that for simplicity, baseline confounders are omitted from the DAG – these are hypothesised to cause all other variables*

*Imputation procedure*

The predictive model contained body mass index (BMI) z-scores at age 11.7 years, General Certificate of Secondary Education (GCSE) and equivalents capped scores, depressive symptoms summary scores, externalising behaviours summary scores (we used the pro-rated scores), school enjoyment summary scores, bullying summary scores, maternal smoking in pregnancy, maternal housing tenure during pregnancy, maternal highest education qualification during pregnancy, maternal age at delivery of the child, and the child's ethnicity. We also included child intelligence quotient (IQ), low birth weight (<2500 g), maternal post-natal depression, maternal marital status during pregnancy, paternal highest education qualification during pregnancy, whether the mother had been homeless during pregnancy, and gestation at delivery. We also included maternal responses to questions on whether child enjoys school (maternal responses at 9.7 years, 11.7 years, and 13.9 years), maternal financial difficulties score during pregnancy, internalising behaviours scores (from five maternal reports between age four years and 13.2 years and a school report from 8.3 years, using pro-rated scores), as well as additional data on BMI z-scores (measures from the clinics visits from age seven years to 15 years), depressive symptoms summary scores (from eight time points between 12.8 to 23.9 years), externalising behaviours scores (from four maternal reports between age four years and 11.7 years using the pro-rated scores), school enjoyment (previous child reports at age 11.2 years), and bullying (from the clinic visit at 10.6 years). Children attended a clinical assessment when they were on average 8 years old and their IQ was assessed using a short from of the Weschler Intelligence Scale for Children (WISC)-III.

Imputation was carried out separately for females and males, with 100 datasets imputed. We used logistic regression to impute binary variables. We used multinomial regression for imputing maternal highest education qualification during pregnancy, paternal highest education qualification during pregnancy, and maternal occupational social class during pregnancy. We used ordinal regression for imputing maternal responses to whether the study child enjoys school. We used linear regression for imputing the BMI measures and the IQ measure. We used predictive mean matching (to one of ten nearest neighbours) for imputing the depression symptom summary scores, externalising behaviours summary scores, school enjoyment summary scores, bullying summary scores, internalising behaviours scores, maternal depression, and maternal financial difficulties scores to make the distribution of the imputed variables consistent to the complete case variables.

*Table S1: Comparison of distributions for imputed data, and unimputed study sample for BMI categories for the female (n = 3,544) and male (n = 3,405) participants*

| | *Females Imputed data* | *Females Observed data (study sample)*[1] | *Males Imputed data* | *Males Observed data (study sample)*[2] |
|---|---|---|---|---|
| *BMI category at 11.7 years (%)*[3] | | | | |
| *(Normal/Underweight)* | *71.4* | *71.8* | *69.1* | *69.8* |
| *(Overweight)* | *13.5* | *13.3* | *13.7* | *13.3* |
| *(Obese)* | *15.1* | *14.9* | *17.2* | *16.9* |

[1] *BMI measures available for 2,749 female participants*

[2] *BMI measures available for 2,588 male participants*

[3] *Children were classified as normal/underweight (BMI z-score <1.04), overweight (BMI z-score ≥1.04 and <1.64, ≥ 85th and <94th percentile) and those with obesity (BMI z-score ≥1.64, ≥95th percentile)(1, 2).*

*Table S2: Comparison of distributions for imputed data, and unimputed study sample for the female participants (n = 3,544)*

| | Percent imputed (%) | Imputed data | Observed data (study sample) |
|---|---|---|---|
| BMI z-score at 11.7 years (mean (SD)) | 22.4 | 0.33 (1.22) | 0.30 (1.20) |
| Externalising behaviours score (average age 13.2 years) (mean (SD)) | 29.1 | 3.86 (3.26) | 3.69 (2.96) |
| Depressive symptoms score (average age 13.8 years) (mean (SD)) | 32.5 | 5.76 (5.76) | 5.65 (4.92) |
| School enjoyment scores (average age 14.2 years) (mean (SD)) | 48.3 | 4.42 (2.05) | 4.32 (1.67) |
| Bullying/Peer victimisation scores (average age 12.8 years) (mean (SD)) | 26.8 | 1.88 (3.06) | 1.83 (2.66) |
| Maternal smoking in pregnancy = 1 (%) | 1.4 | 19.2 | 19.1 |
| Maternal social class (%) | 14.4 | | |
| (I – professional/II - Managerial and technical) | | 33.4 | 34.0 |
| (IIINM - Skilled non-manual/IIIM - Skilled manual) | | 46.4 | 46.8 |
| (IV - Partly skilled/V – Unskilled) | | 20.2 | 19.2 |
| Maternal education (%) | 0.4 | | |
| (Degree) | | 13.2 | 13.3 |
| (A-level) | | 25.3 | 25.3 |
| (O-level) | | 36.2 | 36.2 |
| (vocational) | | 9.6 | 9.6 |
| (none/CSE) | | 15.7 | 15.7 |
| Maternal housing tenure (Council rented/ Housing association rented) (%) | 2.5 | 11.0 | 10.8 |
| Parity (≥1)(%) | 4.1 | 54.4 | 54.5 |
| Maternal age at birth (mean (SD)) | 0.0 | 28.6 (4.53) | 28.6 (4.53) |
| Capped GCSE point score (mean (SD)) | 0.0 | 346.8 (79.45) | 346.8 (79.45) |

*Table S3: Comparison of distributions for imputed data, and unimputed study sample for the male participants (n = 3,405)*

| | Percent imputed (%) | Imputed data | Observed data (study sample) |
|---|---|---|---|
| BMI z-score at 11.7 years (mean (SD)) | 24.0 | 0.44 (1.22) | 0.41 (1.19) |
| Externalising behaviours score (average age 13.2 years) (mean (SD)) | 29.8 | 4.75 (3.68) | 4.53 (3.26) |
| Depressive symptoms score (average age 13.8 years) (mean (SD)) | 35.2 | 4.10 (4.67) | 4.05 (3.81) |
| School enjoyment scores (average age 14.2 years) (mean (SD)) | 57.8 | 4.45 (2.39) | 4.38 (1.69) |
| Bullying/Peer victimisation scores (average age 12.8 years) (mean (SD)) | 29.3 | 1.85 (3.23) | 1.85 (2.90) |
| Maternal smoking in pregnancy = 1 (%) | 1.4 | 19.8 | 19.7 |
| Maternal social class (%) | 12.2 | | |
| (I – professional/II - Managerial and technical) | | 33.0 | 33.5 |
| (IIINM - Skilled non-manual/IIIM - Skilled manual) | | 48.1 | 48.3 |
| (IV - Partly skilled/V – Unskilled) | | 19.0 | 18.2 |
| Maternal education (%) | 0.2 | | |
| (Degree) | | 13.0 | 13.1 |
| (A-level) | | 25.2 | 25.2 |
| (O-level) | | 36.5 | 36.5 |
| (vocational) | | 9.8 | 9.8 |
| (none/CSE) | | 15.4 | 15.4 |
| Maternal housing tenure (Council rented/ Housing association rented) (%) | 2.4 | 10.5 | 10.4 |
| Parity (≥1)(%) | 3.9 | 54.7 | 54.7 |
| Maternal age at birth (mean (SD)) | 0.0 | 29.0 (4.59) | 29.0 (4.59) |
| Capped GCSE point score (mean (SD)) | 0.0 | 325.2 (87.74) | 325.2 (87.74) |

Table S4 – Total causal effect (TCE), natural direct effect (NDE), natural indirect effect (NIE), and proportion mediated of BMI on educational attainment examining the individual mediators and restricting to those with a white British ethnic background from the pooled results for females (n = 3,397) and males (n = 3,263)

| Mediator | Intermediate confounder(s) | TCE coeff (95% CI) | NDE coeff (95% CI) | NIE coeff (95% CI) | Proportion mediated (%) |
|---|---|---|---|---|---|
| Females | | | | | |
| Bullying | - | -3.67 (-5.69, -1.65) | -3.42 (-5.45, -1.39) | -0.25 (-0.58, 0.08) | 6.9 |
| Externalising behaviours | Bullying | -3.67 (-5.69, -1.65) | -2.14 (-4.34, 0.07) | -1.53 (-2.94, -0.13) | 42.1 |
| Depression | Bullying and Externalising behaviours | -3.67 (-5.79, -1.56) | -3.62 (-5.75, -1.50) | -0.05 (-0.28, 0.19) | 1.3 |
| School enjoyment | Bullying, Externalising behaviours, and Depression | -3.67 (-5.86, -1.48) | -3.73 (-5.97, -1.49) | 0.06 (-0.77, 0.88) | N/A |
| Males | | | | | |
| Bullying | - | -3.57 (-5.96, -1.18) | -3.22 (-5.61, -0.84) | -0.34 (-0.73, 0.05) | 9.7 |
| Externalising behaviours | Bullying | -3.57 (-5.96, -1.18) | -2.24 (-4.84, 0.36) | -1.33 (-3.02, 0.36) | 37.5 |
| Depression | Bullying and Externalising behaviours | -3.57 (-5.97, -1.16) | -3.42 (-5.82, -1.01) | -0.15 (-0.58, 0.28) | 4.3 |
| School enjoyment | Bullying, Externalising behaviours, and Depression | -3.57 (-5.94, -1.19) | -3.50 (-5.87, -1.13) | -0.07 (-0.34, 0.20) | 1.9 |

We carried out another set of imputations (m = 100) after restricting to participants with a white British ethnic background (147 females and 142 males were excluded from the analysis). Models were adjusted for maternal age at pregnancy (years), maternal smoking in pregnancy, housing tenure, highest maternal education qualification, maternal social class, and parity. The proportion mediated is not calculated where there is inconsistent mediation, i.e. where the indirect effect is positive and the total effect is negative. The total causal effect 95% confidence intervals differ slightly between the models because of the estimation procedure.

Table S5 – Total causal effect (TCE), natural direct effect (NDE), natural indirect effect (NIE), and proportion mediated of BMI on educational attainment from the sequential causation mediation restricting to those with a white British ethnic background from the pooled results for females (n = 3,397) and males (n = 3,263)

| Mediator(s) | TCE coeff (95% CI) | NDE coeff (95% CI) | NIE coeff (95% CI) | Proportion mediated (%) |
|---|---|---|---|---|
| Females | | | | |
| Bullying | -3.67 (-5.69, -1.65) | -3.42 (-5.45, -1.39) | -0.25 (-0.58, 0.08) | 6.9 |
| + Externalising behaviours | -3.67 (-5.86, -1.48) | -2.51 (-4.83, -0.19) | -1.16 (-2.51, 0.18) | 31.9 |
| + Externalising behaviours + Depression | -3.67 (-5.80, -1.54) | -4.10 (-6.38, -1.83) | 0.43 (-0.94, 1.80) | N/A |
| + Externalising behaviours + Depression + School enjoyment | -3.67 (-5.77, -1.57) | -2.58 (-4.94, -0.22) | -1.09 (-2.65, 0.47) | 30.0 |
| Males | | | | |
| Bullying | -3.57 (-5.96, -1.18) | -3.22 (-5.61, -0.84) | -0.34 (-0.73, 0.05) | 9.7 |
| + Externalising behaviours | -3.57 (-5.94, -1.19) | -2.47 (-5.11, 0.16) | -1.09 (-2.83, 0.65) | 30.8 |
| + Externalising behaviours + Depression | -3.57 (-6.02, -1.11) | -2.62 (-5.25, 0.01) | -0.95 (-2.63, 0.73) | 26.7 |
| + Externalising behaviours + Depression + School enjoyment | -3.57 (-5.95, -1.18) | -2.58 (-5.18, 0.01) | -0.98 (-2.68, 0.72) | 27.6 |

We carried out another set of imputations (m = 100) after restricting to participants with a white British ethnic background (147 females and 142 males were excluded from the analysis). Models were adjusted for maternal age at pregnancy (years), maternal smoking in pregnancy, housing tenure, highest maternal education qualification, maternal social class, and parity. The proportion mediated is not calculated where there is inconsistent mediation, i.e. where the indirect effect is positive and the total effect is negative. The total causal effect 95% confidence intervals differ slightly between the models because of the estimation procedure.

Table S6 Total causal effect (TCE), natural direct effect (NDE), natural indirect effect (NIE), and proportion mediated of BMI on educational attainment examining the overt and relational bullying from the pooled results for the females (n = 3,544) and males (n = 3,405)

| Mediator | TCE coeff (95% CI) | NDE coeff (95% CI) | NIE coeff (95% CI) | Proportion mediated (%) |
|---|---|---|---|---|
| Females | | | | |
| Overt Bullying | -3.49 (-5.57, -1.42) | -3.25 (-5.33, -1.17) | -0.24 (-0.53, 0.05) | 6.9 |
| Relational Bullying | -3.49 (-5.57, -1.42) | -3.43 (-5.51, -1.34) | -0.07 (-0.36, 0.23) | 1.9 |
| Males | | | | |
| Overt Bullying | -4.25 (-6.65, -1.86) | -4.08 (-6.49, -1.67) | -0.18 (-0.45, 0.10) | 4.2 |
| Relational Bullying | -4.25 (-6.65, -1.86) | -4.09 (-6.50, -1.68) | -0.16 (-0.57, 0.25) | 3.8 |

We carried out another set of imputations (m = 100) for analysing the overt bullying and relational bulling separately. These were included in the model instead of the overall bullying summary score. Models were adjusted for maternal age at pregnancy (years), maternal smoking in pregnancy, housing tenure, highest maternal education qualification, maternal social class, and parity

Table S7 – Total causal effect (TCE), natural direct effect (NDE), natural indirect effect (NIE), and proportion mediated of BMI on educational attainment examining the individual mediators excluding participants with Special Education Need record from the pooled results for females (n= 3,308) and males (n = 2,926)

| Mediator | Intermediate confounder(s) | TCE coeff (95% CI) | NDE coeff (95% CI) | NIE coeff (95% CI) | Proportion mediated (%) |
|---|---|---|---|---|---|
| Females | | | | | |
| Bullying | - | -2.94 (-4.93, -0.95) | -2.85 (-4.85, -0.86) | -0.09 (-0.28, 0.10) | 3.1 |
| Externalising behaviours | Bullying | -2.94 (-4.93, -0.95) | -1.81 (-3.99, 0.36) | -1.13 (-2.39, 0.14) | 38.6 |
| Depression | Bullying and Externalising behaviours | -2.94 (-4.91, -0.98) | -2.90 (-4.87, -0.93) | -0.04 (-0.25, 0.17) | 1.3 |
| School enjoyment | Bullying, Externalising behaviours, and Depression | -2.94 (-4.91, -0.98) | -2.75 (-4.76, -0.73) | -0.19 (-0.98, 0.59) | 6.6 |
| Males | | | | | |
| Bullying | - | -2.67 (-5.06, -0.27) | -2.34 (-4.76, 0.07) | -0.32 (-0.71, 0.06) | 12.4 |
| Externalising behaviours | Bullying | -2.67 (-5.06, -0.27) | -1.86 (-4.44, 0.72) | -0.81 (-2.35, 0.74) | 30.5 |
| Depression | Bullying and Externalising behaviours | -2.67 (-4.97, -0.36) | -2.39 (-4.68, -0.09) | -0.28 (-0.75, 0.19) | 10.6 |
| School enjoyment | Bullying, Externalising behaviours, and Depression | -2.67 (-5.03, -0.30) | -2.67 (-5.04, -0.30) | 0.01 (-0.28, 0.29) | N/A |

We carried out another set of imputations (m = 100) for excluding participants who had a record documented in Key Stage 4 of having either Special Education Need (SEN) School Action or a SEN Action Plus (236 females and 479 males excluded from the analysis). Models were adjusted for maternal age at pregnancy (years), maternal smoking in pregnancy, housing tenure, highest maternal education qualification, maternal social class, and parity. The proportion mediated is not calculated where there is inconsistent mediation, i.e. where the indirect effect is positive and the total effect is negative. The total causal effect 95% confidence intervals differ slightly between the models because of the estimation procedure.

Table S8 – Total causal effect (TCE), natural direct effect (NDE), natural indirect effect (NIE), and proportion mediated of BMI on educational attainment from the sequential causation mediation excluding participants with Special Education Need record from the pooled results for females (n= 3,308) and males (n = 2,926)

| Mediator(s) | TCE coeff (95% CI) | NDE coeff (95% CI) | NIE coeff (95% CI) | Proportion mediated (%) |
|---|---|---|---|---|
| Females | | | | |
| Bullying | -2.94 (-4.93, -0.95) | -2.85 (-4.85, -0.86) | -0.09 (-0.28, 0.10) | 3.1 |
| + Externalising behaviours | -2.94 (-4.91, -0.98) | -1.80 (-3.92, 0.32) | -1.14 (-2.39, 0.11) | 39.0 |
| + Externalising behaviours + Depression | -2.94 (-4.89, -1.00) | -2.39 (-4.61, -0.17) | -0.55 (-1.86, 0.76) | 18.9 |
| + Externalising behaviours + Depression + School enjoyment | -2.94 (-4.83, -1.05) | -1.24 (-3.32, 0.83) | -1.70 (-3.14, -0.26) | 58.1 |
| Males | | | | |
| Bullying | -2.67 (-5.06, -0.27) | -2.34 (-4.76, 0.07) | -0.32 (-0.71, 0.06) | 12.4 |
| + Externalising behaviours | -2.67 (-5.03, -0.30) | -2.42 (-5.03, 0.19) | -0.24 (-1.81, 1.32) | 9.1 |
| + Externalising behaviours + Depression | -2.67 (-5.00, -0.33) | -3.38 (-5.90, -0.86) | 0.71 (-0.84, 2.27) | N/A |
| + Externalising behaviours + Depression + School enjoyment | -2.67 (-5.03, -0.30) | -4.36 (-6.96, -1.76) | 1.70 (0.06, 3.33) | N/A |

We carried out another set of imputations (m = 100) for excluding participants who had a record documented in Key Stage 4 of having either Special Education Need (SEN) School Action or a SEN Action Plus (236 females and 479 males excluded from the analysis). Models were adjusted for maternal age at pregnancy (years), maternal smoking in pregnancy, housing tenure, highest maternal education qualification, maternal social class, and parity. The proportion mediated is not calculated where there is inconsistent mediation, i.e. where the indirect effect is positive and the total effect is negative. The total causal effect 95% confidence intervals differ slightly between the models because of the estimation procedure.

References


1. Reilly JJ. Childhood Obesity: An Overview. *Children & Society*. 2007; 21(5): 390-396.
2. Reilly JJ, Summerbell CD, Wilson M, Wilson D. Obesity diagnosis, prevention, and treatment: evidence-based answer to common questions. . *Arch Dis Child*. 2002; 86(6): 392-394.
